# The Queen's Jubilee Hospital

**Published:** 1898-04-30

**Authors:** 


### The Institutional Workshop.

### THE QUEEN'S JUBILEE HOSPITAL.

Sir Henry Burdett was severely taken to task by the Editor of *Lloyd's Weekly Newspaper* for some remarks he made in support of the contention that abuses and unworthiness were not the monopoly of special hospitals. Sir Henry actually spoke as follows :

"I think that another point has been overlooked in criticising the hospital system of London, and it is this. There has grown up of late a much worse class of institution, which has been sprung upon us, and which masquerades in false clothing—I allude to one particular institution, which I name because I consider it a scandal to London. It is the so-called Queen's Jubilee Hospital. This place is not a hospital at all; it is an old house which has been improperly crowded with a greater number of sick patients than it was ever intended to hold of healthy people, and it is conducted on principles which I consider to be dangerous to the public and altogether wrong. I know, from an interview which I had with the chief surgeon of that institution, that he maintains that as he is qualified to treat disease, there is no law against his treating the most serious case in the cellar of the institution if he likes. I think, however, that anyone who tells the people of London that that is a principle which he is prepared to defend is self-condemned, and that we need not trouble ourselves either with him or with his institution. The worst of it is, however, and that is why I mention it, the working classes of the metropolis seem to have an idea that when an institution of this class is attacked by those who can speak with authority and knowledge in the public interest, that then that institution is placed in the position of a martyr, and that it is their privilege and function to defend its existence and to support its continuance. If they only knew what many of you members of the medical profession know, they would realize that in doing that, in giving support to institutions of this class, they are unwittingly, but no less certainly, fostering a great danger to poor people who may be struck down by accident, or who may suffer from serious disease. That is the evil and danger of the attitude of the working classes towards hospitals so-called—mis-called in my judgment —like that to which I have ventured to allude, in order that I may show that it is wrong to suppose that bad management, or the unnecessary uprising of what are known as one-man institutions, are confined to special hospitals, for the Queen's Jubilee Hospital claims to be a general hospital. I can only hope that the people who reside in the neighbourhood of Brompton, and who wish to see that the poor of the metropolis are properly provided with medical assistance, will go down to the building and will insist upon going over it, so that they may judge of it as it is for themselves. When I asked to be permitted to make a plan of the building and to show on that plan how the beds were placed in the wards, and what was the height of the wards, and what must necessarily be the condition of the atmosphere, I was absolutely refused that permission. No words of mine can be more condemnatory than that fact, and I hope, though I am sorry to see that the institution has had the support of an influential paper like *Lloyd's*, that something may be done either to close the institution, or at any rate to have it pulled down, and a proper building erected in its place. Perhaps I have taken up too much of your time in dealing with this point, but I have done it as a mere act of justice to special hospitals as a whole, in order to show that they have not the monopoly of mismanagement, but that certain people who have set out to form what they please to call a general hospital, have much more to be condemned for than the founder of any special hospital."

*Lloyd's* maintains that to speak thus was to push one hospital by condemning another. Its Editor further adds: "If the Hospital Saturday Fund is doing wrong by contributing year after year to the Jubilee Hospital, let the reason be made known," and further, "we cannot accept the condemnation of one man, however eminent." There is a sweet reasonableness about this suggestion which has induced us to ask Dr. Solomon Smith to visit the Queen's Jubilee Hospital this week, and to give an account of it as he actually found it. The following report by Dr. Smith will surely convince every intelligent reader that Sir Henry Burdett's contention that the so-called Queen's Jubilee Hospital should be pulled down and rebuilt, or else that it should be closed finally, is justified on the facts and in the public interest. At any rate, in face of this report it must be generally admitted that, unless the Editor of *Lloyd's Newspaper* is completely dominated—as he is credited with being —by his friendship for Mr. Benham, whose interests may demand that this establishment should be kept open at all costs, he must on the facts agree that the present building cannot be properly regarded as a hospital, and that it shall cease to be used for hospital purposes.

### The Queen's Jubilee Hospital as Our Commissioner found it on April 21st, 1898.

The Queen's Jubilee Hospital is a small two-storied house standing a little way back from the road, from which it is separated by a gravelled fore-court. Looking at the house from the road, one sees on the ground floor a front door and one window on each side of it, and on the floor above three windows. The window on the right belongs to the board-room, behind which is a little room into which I did not see. The whole thickness of the house, however, from front to back on the right side of the entrance-hall, I reckon at about 19 ft. The window on the left of the front door belongs to the consulting-room, in which out-patients are seen and operations are performed. This is a room about 14 ft. wide by 15 ft. long, and behind it is the out-patient waiting-room, carrying the house back another 15 ft., where it ends by steps leading down into the garden. It will thus be seen that the house extends further back on the left side of the door than on the right—*i.e.*, about 30 ft. on the one side, not including walls, and between 18 ft. and 19 ft. on the other. Now, still looking at the plan from the same point of view, it would seem that at some period in the history of the house an annexe has been added to it, on the right side of the projection formed by the waiting-room, containing a lavatory and w.c. Whether or no this was built at the same time as the rest of the house—so far as concerns the basement and ground-floor—I cannot say, but certainly as regards the upper floor, where a bath-room and water closet are placed, it is an addition, and is thus outside the main walls of the house. As regards these closets, I would say that the room upstairs contains a w.c., a bath, and a sink, together with the hot-water cistern, that is one w.c. for the patients of both sexes in the three rooms upstairs. It is a long strip of a room, perhaps about 4 ft. 6 in. wide, lighted by a window and by a skylight. The w.c. is of the wash-down pattern; the flush seems efficient, and the soil-pipe is outside the wall and is carried up above the roof. The closet on the ground-floor is under the one upstairs, and is entered through a lobby, lighted by a window, and having in it a lavatory basin. The w.c. also discharges outside into a soil-pipe carried above the roof. The flush was in good working order, but the pan was not in very good condition. In the basement was a w.c., which I can only describe as a foul closet. The pan was dirty, and I could see no sign of the soil-pipe being carried up. This closet is open for use by the out-patients, and is, in fact, close to where they wait for their medicines. Connected with the drain at the back of the house is a man-hole or inspection chamber, which I did not open, into which, I presume, these soil-pipes discharge, and in the front of the house there is another inspection hole, the drain between the two passing under the house. To return now to the house itself. The consulting-room is about 14 ft. wide by 15 ft. long. Like the other rooms, it has a deal floor, a painted dado, and distempered walls above. One corner is curtained off as an undressing or examining place. The room is provided with an earthenware sink, properly trapped, an operating table, a glass-topped instrument table, a steriliser for instruments, and an ordinary wooden cupboard for instruments, the supply of which is not large, but is said to be supplemented by the surgeons bringing their own when required. Behind the consulting-room is the waiting-room, about 15 ft. by 18 ft. This is entered from the garden by some wooden steps. In this room at the time of my visit were 27 women and children. Both these rooms are about 10 ft. high. The dispensary is in the basement. The room is 12 ft. 6 in. by 11 ft. 6 in., or thereabouts. It is provided with a sink, and with a gas stove on which a pan or kettle can be boiled. In size and in shelf space this room would appear sufficient for its purpose, but evidences of both poverty and neglect are strikingly abundant. I especially wish to avoid saying anything which should appear to criticise the treatment of the patients, but, so far as the appliances for dispensing are concerned, it appears to me that this institution is lamentably deficient. The place also in which the patients stand while waiting for their medicines seems quite unfit for delicate people in cold weather. In addition to the dispensary, the basement contains a kitchen, a scullery, a larder, and some other cellaring.

Going upstairs, we find on the right the female ward, which is a room containing four beds and measuring approximately 18 ft. 3 in. long by 10 ft. 3 in. wide, having in addition a bay by the door measuring 6 ft. 4 in. by 4 ft. 4 in., and is 9 ft. high. This gives us by rough measurement, without deducting anything for furniture, a total cubic capacity of 1,927 feet, which, divided by the number of beds in the room, works out to the surprising figure of only 481·8 cubic feet per bed. It should be mentioned that there is a ventilator in the ceiling leading into the false roof. One of the beds in this room is a short one, intended only for a child.

In the male ward there are four beds. The dimensions of the room are roughly 13 ft. 6 in. by 18 ft., by 9 ft. high; giving a cubic capacity of 2,187 ft., or of 546·7 ft. per bed. There is a ventilating opening in the ceiling leading into the false roof. Each of these wards has a fireplace.

At the top of the staircase, and occupying the space over the hall is a single bedded ward, a little room 7 ft. by 13 ft. Taking the height of this room as the same as the others, that would give a cubic capacity of 819 ft. This room, however, has no fireplace, and for its heating in winter has to depend upon a small condensing stove, and for ventilation on the door and window.

The other rooms upstairs are a bed-sitting-room for the secretary and a small two-bedded room for the nurses.

Passing now from what I saw to what I was told, we come to the question of the staff. Including the house surgeon (non-resident), who also does the dispensing, I was told that six medical men see patients; that there is someone in attendance every day; that each medical officer takes any case that comes, but that arrangements are made by which, at second and all subsequent visits, the cases are sorted out according to certain specialties, so as to keep certain days and certain medical officers for particular classes of disease. As to management, I was told of the existence of a committee of management meeting once a month, a weekly board, and a finance committee, each meeting once a week, and sub-committees as required.

The following persons are employed: One lady acting-secretary, who lives in the house; two nurses, one of whom, being the night nurse, sleeps outside; one probationer, who lives in the house; one ward or housemaid, who lives in the house; one cook, who lives in the house; one out-patient clerk, who lives outside; one surgery porter, who lives outside; one errand boy, who lives outside. It is to be noted that the cook and the housemaid have no bedroom, but that they sleep in the out-patient waiting-room, their beds being removed each morning.

I was unable to obtain any very definite information as to the number of patients treated in the year, either as in-patients or out-patients; but I was shown the records of the daily attendances, from which it would appear that the new cases are classified as ticket cases and casualty cases, and that a daily entry is also made of the total number that attend, whether as new cases or repeats; and I was informed that the total of the attendances of all sorts was between three and four hundred a week. It was clear, however, that the number varied greatly from day to day. I was told that some time ago a couple of hundred patients might have been seen in a day, but not lately, the numbers recently not having ever exceeded 80 or 90 in a day. Certain arrangements, however, are made in regard to hours of attendance—the men, for example, coming at a different hour from the women—by which the crush upon the waiting-room is lessened, and although, compared with proper hospitals, the accommodation provided is meagre, I am not prepared to raise any objection to the physical conditions under which these out-patients are attended to, as they existed on the day of my visit, except so far as concerns the place in which they wait for their medicines. It is, however, as a hospital, not as a dispensary, that this institution appeals to the public, and must, therefore, be considered by me.

Now, I would wish to be in every way perfectly fair to every form of charity, and to put entirely on one side those personal questions which have arisen in regard to the conduct of this institution; but I would ask, Is it right that patients should be taken for serious surgical treatment into a house which only offers in one of its wards between four and five hundred cubic feet of space per bed, and in another between five and six hundred—in both cases below the minimum enforced in every barrack in the country, and far below what is provided in many workhouse infirmaries? And I would further ask, Is it right that such an institution as I have, I hope, honestly and fairly described should go before the public as a hospital, and should as such ask for support, when the standard of the provision it makes for its patients differs in so striking a degree from that which obtains in those great institutions to which the giving public rightly attaches the honoured name of "Hospital"?

Of course, much depends upon point of view. To anyone accustomed to the wards of our great hospitals, or even of many of our workhouse infirmaries, there is something positively shocking in calling this squalid little house a hospital. Yet, as I say, there is much in point of view; and I am free to confess, as one who has had many opportunities of studying many phases of human life, that, as the wards of this institution appeared on the occasion of my visit—that is, only half full—patients were likely to be better off in them than they would be in a certain proportion of the homes of the very poor. This, no doubt, is the plea which will be entered for the continuance of this institution. But it seems to me that this is not the point. If there were no other places for the sick, if there were no other hospitals, and no other infirmaries, perhaps something might be said for opening institutions whose standard was lower. But all these institutions do exist; it is notorious that beds in really well-appointed hospitals are standing empty at the present time for want of money; and, under such circumstances, it seems both a folly and a waste to support such an institution as the Queen's Jubilee Hospital when so much more good can be done for the money by the real hospitals which exist on every side.

